# Economic Evaluation of Nutrition-Sensitive Agricultural Interventions to Increase Maternal and Child Dietary Diversity and Nutritional Status in Rural Odisha, India

**DOI:** 10.1093/jn/nxac132

**Published:** 2022-06-10

**Authors:** Hassan Haghparast-Bidgoli, Helen Harris-Fry, Abhinav Kumar, Ronali Pradhan, Naba Kishore Mishra, Shibananth Padhan, Amit Kumar Ojha, Sailendra Narayan Mishra, Emily Fivian, Philip James, Sarah Ferguson, Sneha Krishnan, Meghan O'Hearn, Tom Palmer, Peggy Koniz-Booher, Heather Danton, Sandee Minovi, Satyanarayan Mohanty, Shibanand Rath, Suchitra Rath, Nirmala Nair, Prasanta Tripathy, Audrey Prost, Elizabeth Allen, Jolene Skordis, Suneetha Kadiyala

**Affiliations:** University College London, Institute for Global Health, London, United Kingdom; London School of Hygiene & Tropical Medicine, London, United Kingdom; Digital Green, New Delhi, India; Digital Green, New Delhi, India; Voluntary Association for Rural Reconstruction and Appropriate Technology (VARRAT), Kendrapada, Odisha, India; Voluntary Association for Rural Reconstruction and Appropriate Technology (VARRAT), Kendrapada, Odisha, India; Ekjut, Chakradharpur, Jharkhand, India; Voluntary Association for Rural Reconstruction and Appropriate Technology (VARRAT), Kendrapada, Odisha, India; London School of Hygiene & Tropical Medicine, London, United Kingdom; London School of Hygiene & Tropical Medicine, London, United Kingdom; Devoted Health, Waltham, MA, USA; Jindal School of Environment and Sustainability, OP Jindal Global University and ETCH Consultancy Services, Mumbai, Maharashtra, India; Tufts University Friedman School of Nutrition Science and Policy, Boston, MA, USA; University College London, Institute for Global Health, London, United Kingdom; SI Research & Training Institute, Inc. Arlington, VA, USA; SI Research & Training Institute, Inc. Arlington, VA, USA; SI Research & Training Institute, Inc. Arlington, VA, USA; DCOR Consulting Pvt. Ltd. Bhubaneshwar, India; Ekjut, Chakradharpur, Jharkhand, India; Ekjut, Chakradharpur, Jharkhand, India; Ekjut, Chakradharpur, Jharkhand, India; Ekjut, Chakradharpur, Jharkhand, India; University College London, Institute for Global Health, London, United Kingdom; London School of Hygiene & Tropical Medicine, London, United Kingdom; University College London, Institute for Global Health, London, United Kingdom; London School of Hygiene & Tropical Medicine, London, United Kingdom

**Keywords:** Nutrition-sensitive agriculture, Cost–consequence analysis, Participatory learning and action, Women's groups, Dietary diversity, Maternal and child nutrition, India

## Abstract

**Background:**

Economic evaluations of nutrition-sensitive agriculture (NSA) interventions are scarce, limiting assessment of their potential affordability and scalability.

**Objectives:**

We conducted cost–consequence analyses of 3 participatory video-based interventions of fortnightly women's group meetings using the following platforms: *1*) NSA videos; *2*) NSA and nutrition-specific videos; or *3*) NSA videos with a nutrition-specific participatory learning and action (PLA) cycle.

**Methods:**

Interventions were tested in a 32-mo, 4-arm cluster-randomized controlled trial, Upscaling Participatory Action and Videos for Agriculture and Nutrition (UPAVAN) in the Keonjhar district, Odisha, India. Impacts were evaluated in children aged 0–23 mo and their mothers. We estimated program costs using data collected prospectively from expenditure records of implementing and technical partners and societal costs using expenditure assessment data collected from households with a child aged 0–23 mo and key informant interviews. Costs were adjusted for inflation, discounted, and converted to 2019 US$.

**Results:**

Total program costs of each intervention ranged from US$272,121 to US$386,907. Program costs per pregnant woman or mother of a child aged 0–23 mo were US$62 for NSA videos, US$84 for NSA and nutrition-specific videos, and US$78 for NSA videos with PLA (societal costs: US$125, US$143, and US$122, respectively). Substantial shares of total costs were attributable to development and delivery of the videos and PLA (52–69%) and quality assurance (25–41%). Relative to control, minimum dietary diversity was higher in the children who underwent the interventions incorporating nutrition-specific videos and PLA (adjusted RRs: 1.19 and 1.27; 95% CIs: 1.03–1.37 and 1.11, 1.46, respectively). Relative to control, minimum dietary diversity in mothers was higher in those who underwent NSA video (1.21 [1.01, 1.45]) and NSA with PLA (1.30 [1.10, 1.53]) interventions.

**Conclusion:**

NSA videos with PLA can increase both maternal and child dietary diversity and have the lowest cost per unit increase in diet diversity. Building on investments made in developing UPAVAN, cost-efficiency at scale could be increased with less intensive monitoring, reduced startup costs, and integration within existing government programs. This trial was registered at clinicaltrials.gov as ISRCTN65922679.

## Background

There is strong evidence on the impacts and cost-effectiveness of nutrition-specific interventions (1–4), particularly in settings with high undernutrition burdens, such as India. However, to achieve the Sustainable Development Goal (SDG) targets (5) to end hunger and undernutrition (SDG 2), multisectoral approaches are needed to address the underlying causes of undernutrition (6). In rural areas of low- and middle-income countries, where the burden of undernutrition is highest and smallholder farming provides a major source of nutrition and income (7), the agriculture sector could provide “nutrition-sensitive” interventions that improve both nutrition and agriculture outcomes simultaneously..

Trials of nutrition-sensitive agriculture (NSA) interventions have shown that these interventions may improve dietary outcomes, with results suggesting that the implementation of these interventions at scale may be a policy option. Of the 8 trials that tested the effects of NSA interventions on minimum dietary diversity of children, 4 trials showed a significant increase, although neither of the 2 NSA trials measuring impacts on maternal dietary diversity showed an effect (8). However, our ability to recommend upscaling of NSA interventions is constrained by a lack of data on their costs, and therefore a lack of evidence regarding their value for money (6, 9, 10). So far, the few economic evaluations (the family of evaluation types that relate costs and impacts) of NSA interventions that do exist come from sub-Saharan Africa (10–12)—none come from South Asia (13, 14). Policymakers need this evidence to prioritize and justify their investments, particularly in resource-constrained settings in South Asia. Additionally, economic evaluations may be more needed for multisectoral interventions such as NSA, to garner support from the multiple (traditionally separate) sectors involved and justify the efforts required to enable collaboration (15).

One reason for the lack of NSA economic evaluations is that traditional methods developed for single-sector interventions are unsuitable for multisectoral interventions. Cost–benefit analyses (which give cost per economic value of aggregate benefits) rely on too many assumptions to compute the economic value of dietary and agricultural outcomes. Cost-effectiveness analyses (which estimate costs per natural unit of an outcome or a composite health measure) give misleadingly high estimates (16). For interventions designed to affect a range of health and non-health outcomes, assigning all intervention costs to a single outcome creates an erroneous impression that the cost per unit of improvement is prohibitively high. Cost–consequence analyses (where outcomes are reported alongside disaggregated costs) offer a transparent approach that allows policymakers to weigh the evidence for themselves. Cost–consequence analyses are recommended for multisectoral interventions with multiple health and nonhealth effects (17, 18), ideally alongside cost-effectiveness analysis.

Here, we present the economic evaluation results of the UPAVAN trial, conducted in Odisha state, India, between 2016 and 2020 (8, 19). The trial aimed to test the nutritional and agricultural impacts of 3 video-based participatory NSA interventions, each compared with a control arm. The specific objectives of the economic evaluations of the UPAVAN study were the following: *1)* describe intervention coverage and participation; *2*) estimate the program cost and cost efficiency (cost per participant) of implementing the interventions; *3*) estimate societal costs of the interventions; *4*) conduct a cost–consequence analysis to present the costs alongside the effects of the interventions and estimate cost per unit increase in outcome per intervention; *5*) examine effects of uncertain parameters, assumptions, and potential scenarios on intervention costs;, and *6*) estimate the cost of delivering the interventions at scale and affordability of scale-up across rural Odisha.

## Methods

### Overview of the UPAVAN trial study design

Detailed descriptions of the UPAVAN interventions, study design, and impacts are reported elsewhere (8, 19, 20). In brief, the UPAVAN trial was a 4-arm, cluster randomized controlled trial, implemented in 4 administrative blocks (Patna, Keonjhar Sadar, Harichandanpur, and Ghatgaon) in the Keonjhar district, Odisha, India. One or 2 villages and surrounding hamlets were defined as a cluster, to ensure a minimum population of 800 per cluster. Stratified block random assignment was used to allocate 148 clusters to 4 trial arms (3 intervention arms and 1 control arm), giving 37 clusters per arm. Allocation of the clusters was stratified by distance to the nearest town (<10 km or ≥10 km) and the proportion of Scheduled Tribe or Scheduled Caste [historically disadvantaged households: low: 30%, medium: 30–70%; high : >70%] (8, 19), giving 6 strata in total.

Both cluster and individual-level informed consent were obtained for participation in the trial and surveys. Ethics approval for the trial was obtained from the Odisha government's Institutional Review Board, Research and Ethics Committee, Department of Health and Family Welfare, Government of Odisha, and the LSHTM Interventions Research Ethics Committee.

### Study setting

The Keonjhar district has an estimated population of 1.8 million residents, 86% of whom reside in a rural setting (21). Of this population, 44% belonged to the Scheduled Tribes and 12% to Scheduled Castes (21)—historically the most disadvantaged socioeconomic groups in India. The prevalence of maternal and child undernutrition in Keonjhar is among the highest in India. In Keonjhar during 2015–2016, 30% of women (age 15–49 y) were underweight and 40% were anemic (13), and of children aged <5 y, >40% were stunted and 19% were wasted (13).

### UPAVAN interventions

Interventions were implemented by the Voluntary Association for Rural Reconstruction and Appropriate Technology (VARRAT), a nongovernmental organization in Odisha, with technical support from Digital Green, Ekjut, John Snow Inc. Research and Training Institute (JSI RTI), London School of Hygiene & Tropical Medicine (LSHTM), and University College London (UCL). The trial was evaluated by LSHTM, UCL, and DCOR Consulting Pvt. Ltd.

**FIGURE 1:**
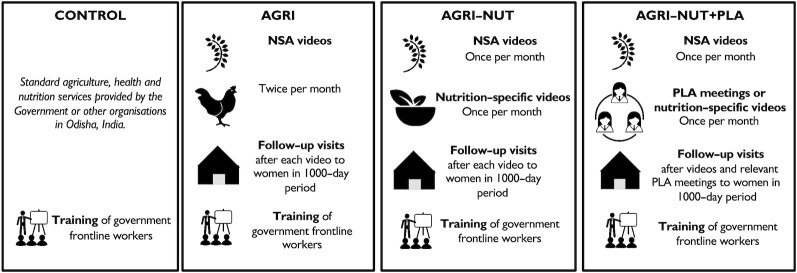
Overview of components delivered across UPAVAN interventions and control arms, adapted with permission from Kadiyala et al. (2018) (19). AGRI, Nutrition-sensitive agriculture intervention; AGRI-NUT, Nutrition-sensitive and nutrition-specific agriculture intervention; AGRI-NUT + PLA, Nutrition-sensitive and nutrition-specific agriculture intervention using the Participatory Learning and Action approach; PLA, Participatory Learning and Action; UPAVAN, Upscaling Participatory Action and Videos for Agriculture and Nutrition.

The 3 UPAVAN interventions have been described in detail elsewhere (19, 20) and are briefly described here, with an overview in **Figure 1**. Each intervention included 2 main components: a fortnightly women's group meeting (the content of which varied between interventions) and a follow-up home visit to each group member after each meeting. The interventions worked with women's self-help groups (SHGs)—an existing platform involved in savings and credit activities. The interventions were implemented for 32 mo, from March 2017 to October 2019.

#### Nutrition-sensitive agriculture intervention (AGRI)

In this arm, fortnightly women's groups viewed and discussed NSA videos, following a participatory video approach designed by Digital Green (22). The participatory video approach had 4 steps: *1*) local implementers identified relevant NSA practices to include in videos, *2*) local videographers filmed farmers and other community members demonstrating or discussing the practices, *3*) local facilitators screened the videos in group meetings and facilitated discussions, and *4*) facilitators conducted follow-up home visits to pregnant women and mothers of children aged 0–23 mo who participated in the group meetings. NSA videos covered the main themes, following UPAVAN's theory of change (5, 19), of increasing production and diversity of nutritious or income-generating foods, increasing women's decision-making power in agricultural activities, and reducing workloads for pregnant and breastfeeding women.

Quality assurance and monitoring were embedded in the participatory video process. Facilitators kept registers to track attendance, whether participants were pregnant or had a child aged 0–23 mo, and whether a government frontline health and nutrition care provider attended the meeting. During home visits, facilitators completed forms to record participants adoption and/or recall of practices promoted in the previous meeting.

#### Nutrition-sensitive and nutrition-specific agriculture intervention (AGRIAGRI-NUT)

This arm used the same participatory video approach as in the AGRI arm, but videos covered both NSA and nutrition-specific topics. The AGRI-NUT group videos received were half NSA videos shown in the AGRI arm and half nutrition-specific videos covering topics on infant and young-child feeding practices and maternal diets.

#### AGRI-NUT + Participatory Learning and Action

This arm used the same participatory video approach as in the AGRI arm but integrated nutrition-specific meetings that followed a Participatory Learning and Action (PLA) approach (using half the NSA videos shown in the AGRI arm and half PLA meetings). In the PLA meetings, the groups followed a 4-phase PLA cycle in which they performed the following tasks: *1*) learned about and prioritized nutrition problems; *2*) discussed and prioritized the causes, effects, and locally feasible strategies to address these problems within their groups and the wider community; *3*) implemented the identified strategies; and *4*) informally evaluated the results of their actions and made future plans. The PLA meetings were either interactive discussions without videos (using participatory techniques such as voting, storytelling, and games), or participatory videos on nutrition-specific topics that were developed as part of the PLA process. Therefore, the nutrition-specific videos in the PLA arm were different from those in the AGRI-NUT arm.

#### Control

Those in the control arm (and intervention arms) received standard government services. In addition, the government frontline health and nutrition workers in all 4 arms received 2 d of training on maternal, infant, and young child nutrition.

### Evaluating coverage and participation

**FIGURE 2:**
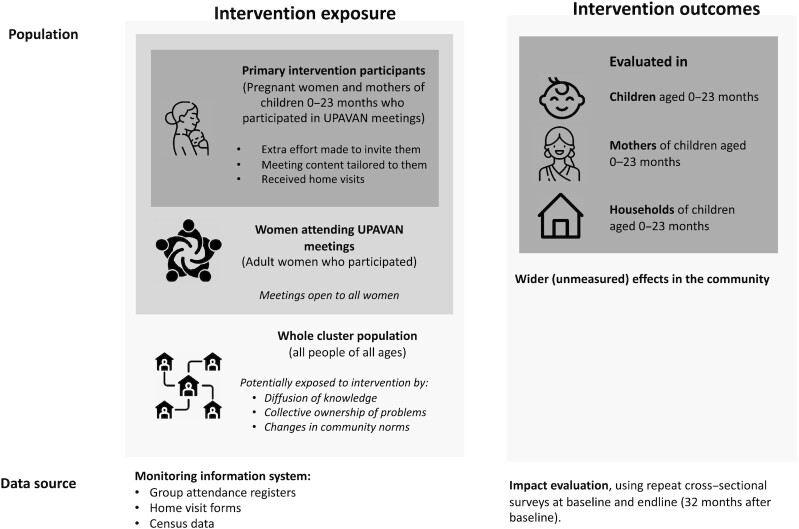
Target populations of UPAVAN interventions. UPAVAN, Upscaling Participatory Action and Videos for Agriculture and Nutrition.

**Figure 2** describes the populations aimed for inclusion according to the interventions (intervention exposure) and benefit (intervention outcomes).

The UPAVAN interventions were primarily designed to include pregnant women and mothers of children aged 0–23 mo (primary intervention participants) and to benefit children aged 0–23 mo, their mothers, and their households. Therefore, we primarily report coverage and cost-efficiency in terms of the former, and impacts (described in the next section) on the latter.

Coverage was assessed as whether or not primary intervention participants attended ≥1 group meeting and received ≥1 home visit, based on monitoring data recorded by group facilitators (registered with records on 46,327 meetings and forms on 149,585 home visits). We also assessed coverage in terms of all participating women, defined as women of any age who attended ≥1 group meeting according to group registers, because all women of all ages in the intervention clusters were eligible to participate in UPAVAN interventions, and the SHG platform that UPAVAN worked with includes women of all ages.

Participation is given as: total group meetings and home visits attended by primary intervention participants; group meetings attended by all women; and total “points of contact,” the sum of group meeting attendance (all women) and home visits.

### Evaluating consequences (trial impacts)

We had 2 primary outcomes. The first outcome was child dietary diversity, measured as the percentage of children aged 6–23 mo consuming ≥4 of 7 food groups in the previous 24 h, using the WHO-defined food groups (23). The second primary outcome was BMI (measured as kg/m^2^) of nonpregnant, nonpostpartum (gave birth >42 d previously) mothers of these children. Secondary outcomes were maternal dietary diversity, measured as percentage of mothers consuming ≥5 of 10 food groups in the previous 24 h using FAO-defined food groups (24), and percentage of children with a weight-for-height -score <–2 SD of the WHO growth standards median (25). The trial was powered for the 2 primary outcomes to give a target sample size of 4736 mother–child pairs (1184 per arm) at baseline and again at endline. Other outcomes on health, women's empowerment, food security, and agricultural production were prespecified and are given in **Supplemental Material Table S1**.

The impact of the interventions was evaluated on children aged 0–23 mo and their mothers (and their households for household-level indicators). The impact evaluation used randomly selected samples of eligible households from each cluster at baseline and endline. Households were eligible if they contained a child aged 0–23 mo with no disability affecting anthropometric measurements, and the child's primary caregiver had no disability impairing their participation in the surveys and had been resident in the household for at least half a year before data collection.

Impacts were analyzed using intention-to-treat analysis. The analyses were cross-sectional, assessing outcomes in each intervention arm compared with the control arm at endline. The analyses adjusted for baseline measures by including all individuals at each timepoint linked by cluster, and outcomes were analyzed using separate generalized estimating equations to account for clustering. Adjusted analyses also included distance to the nearest town and proportion of Scheduled Tribe or Scheduled Caste households as covariates.

Results of the UPAVAN impact evaluation are reported elsewhere (8). In this paper, we have presented adjusted effects on all prespecified outcomes that were statistically significant from control; these comprise the “consequences” in our cost–consequence analysis.

### Evaluating costs

**FIGURE 3:**
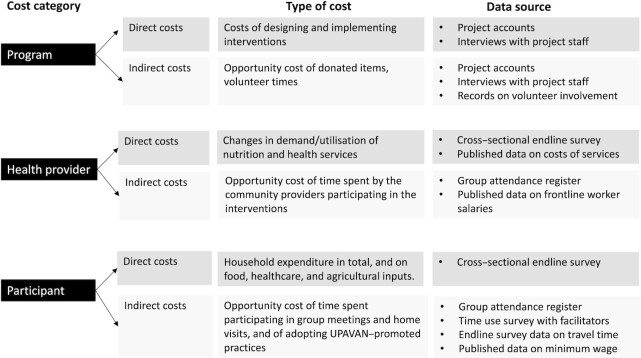
Conceptual framework for cost analysis of UPAVAN interventions, adapted with permission from Haghparast-Bidgoli et al. (2019) (26).

The full cost methodology is presented in the economic evaluation protocol (26) and is summarized in this section and **Figure 3**.

Economic costs of the interventions were estimated from a program perspective (i.e., costs incurred by the implementing agencies) and a societal perspective (i.e., costs to program implementers, the government health system, and program participants). Economic costs refer to direct financial costs plus indirect costs such as value of donated items or time (opportunity) costs of participating. All direct and indirect cost types, including program and societal costs, as well as the data sources and assumptions used for calculating costs, are given in **Supplemental Material Table S2**.

The time horizon for cost analysis was 41 mo, including a 9-mo startup period and 32-mo intervention implementation. Activities during the startup period included recruitment and training of group facilitators and their supervisors, community sensitization activities, and development of video content and PLA meeting plans.

#### Program costs

A combination of activity-based costing (27), expenditure approach, and ingredient approaches (28) were used to estimate program costs. First, the intervention components and associated main activities were identified and defined as cost centers. Then, data on quantity and costs (or estimated value of resources, in case of donated items) were collected and allocated to these cost centers. To estimate the direct costs of designing and implementing the interventions, we collected financial cost data from all UPAVAN partner expenditure records or project accounts. For indirect costs, we identified donated items and volunteered time through interviews with all project staff and estimated their opportunity cost (value) using the current market value for donated items (28–30) and staff monthly salaries for volunteered time. Donated items were mainly video making and editing equipment used by the implementing partner. Volunteered time mainly included unpaid time contributed to the design and adaptation of intervention materials, as well as quality assurance by technical partners.

Cost data were collected using data capture tools (**Supplemental Material–data collection tools**) designed for the project. All data collected were entered into a customized excel-based costing tool, adapted from the costing tool developed by the UCL Centre for Global Health Economics (http://www.ighe.org) for analysis. The tool categorizes the costs based on the following line items: staff, materials, capital, joint costs (shared by several activities such as field travel and partner meetings), and overheads. the tool also categorizes costs based on the intervention component: AGRI, NUT, PLA, training of government frontline health and nutrition care providers, quality assurance and monitoring, or partner coordination, and implementation stage (startup or implementation). Staff costs were allocated to intervention components using data from staff time–use surveys collected through interviews with all project staff (full descriptions of line items, intervention components, and implementation phases are given in **Supplemental Material Table S3**). The same allocation rule was applied to allocate nonstaff joint costs to intervention components. Capital costs were annualized based on the estimated lifetime of each item, using a discount rate of 3%. We included contributions of the international technical (JSI RTI) and research partners (LSHTM and UCL) in developing and supporting interventions, but not research costs.

#### Costs to government health system

We measured direct financial costs and indirect opportunity costs incurred by primary health centers and government frontline health and nutrition care providers [i.e., Anganwadi workers (AWWs), accredited social health activists (ASHAs), and auxiliary nurse midwives (ANMs)].

Direct costs were based on any increase in the use of health and nutrition services (in the past 6 mo), as determined by comparing percentages of households using any health or nutrition services in each intervention relative to control in the endline survey, and published data on unit cost to health centers for providing those services (31–36).

We estimated the indirect costs as the opportunity costs of government frontline health and nutrition care providers participating in the interventions. This calculation was based on group attendance by government frontline health and nutrition workers (assessed from facilitator registers), mean durations of meetings and travel times to meeting locations (from the endline survey), and published monthly salaries of the government frontline health and nutrition care providers (Supplemental Material Table S2).

#### Costs to participants and their households

We estimated opportunity costs to participants of attending dissemination meetings and follow-up home visits by using data on the total number of group attendants and follow-up home visits per intervention arm (from facilitator registers and home visit forms); mean durations of group meetings and home visits, and mean travel time to the meeting location (collected in the UPAVAN endline survey); and the minimum daily wage of an agricultural worker in Odisha state (303 Indian Rupees or US$4.31) (37) (Supplemental Material Table S2).

We estimated cost to households of adopting practices promoted in UPAVAN as any differences in the following expenditures: seeking healthcare (out-of-pocket fees and transport costs paid for child and maternal healthcare from public, private, or informal healthcare providers), agricultural inputs, food costs, and nonfood costs. These costs were estimated as the mean differences between each intervention and the control arm, using the expenditure survey in the UPAVAN endline survey.

### Analysis

Our results are presented in the form of cost–consequence analysis, by tabulating disaggregated costs and outcomes of the interventions compared with the control arm. We selected a program perspective as the base case to reflect the potential budget impact of adopting the intervention. The societal perspective presents the full costs of implementing the interventions.

All costs, including unit costs obtained from published studies, were adjusted for inflation using the Indian Consumer Price Index (38) and converted to 2019 US$, using the exchange rate of 70.42 (39) for the costs to Indian partners (in Indian Rupees), and 0.78 (39) for costs to the UK partners (in British Pounds Sterling, GBP). In addition, costs were discounted at 3% per y, as recommended by WHO-CHOICE (World Health Organization CHOosing Interventions that are Cost-Effective) (40) and the Gates/iDSI Reference Case for Economic Evaluation (41). Our study follows the Consolidated Health Economic Evaluation Reporting Standards (CHEERS) (42).

We calculated total costs and mean annual costs of the interventions. Mean annual costs enable comparisons with other interventions that run for different durations. Mean annual costs were calculated as the sum of startup and implementation costs, divided by 41 mo (the costing time horizon), and multiplied by 12, so they divide startup costs equally across each year. We decomposed the total costs of the interventions and presented these as a share of line-item (i.e., staff, materials, capital, other), intervention component (i.e., NSA videos, nutrition-specific videos, PLA, quality assurance, coordination, frontline worker training), and implementation phase (i.e., startup and implementation).

To estimate the cost-efficiency of the interventions, we calculated the total or annual costs of each intervention per primary intervention participant. We also estimated cost per total point of contact (sum of group attendees and total home visits). We also estimated cost per unit increase in maternal and child dietary diversity scores (i.e., 2 statistically significant primary and secondary outcomes) for each intervention arm, compared with the control arm. They were calculated as mean increase in outcome divided by cost per primary intervention participant.

We conducted a number of univariate sensitivity analyses that vary 1 parameter at a time, and scenarios for potential intervention costs at scale. In sensitivity analysis, we examined the impact of 2 uncertain assumptions: we altered the allocation rule for dividing the costs of the nutrition-specific component between the AGRI-NUT and AGRI-NUT + PLA arms (from a 75:25 rule to a 90:10 rule), and we varied the discount rate from 0% to 6% (40, 41). In scenario analyses, we tested impacts of 3 scenarios that we believe will be relevant when interventions are implemented at scale: reducing startup costs by 50%, reducing costs of monitoring by 25% and 50%, and replacing international staff costs with local staff costs. A detailed description of sensitivity and scenario analyses are presented in the **Supplemental Material - Sensitivity and scenario analyses**.

Finally, we estimated the potential cost of delivering the AGRI-NUT + PLA intervention to all rural populations in Odisha state, by dividing the total intervention cost by the total population in the intervention clusters (based on census data) and multiplying by the total rural population of Odisha.

## Results

### Trial coverage and participation

UPAVAN's coverage and participation are described in **Table 1**. Throughout the 32-mo implementation period, the UPAVAN interventions covered a mean of 4567 primary intervention participants, and 9367 women of any age, per intervention arm. So, approximately one-half of participants were pregnant women and mothers of children aged 0–23 mo, and 17% of the total population directly participated in UPAVAN intervention activities, per arm. Assuming an average of 5 members per household (and 1 participant per household, and that household members discussed the interventions), the interventions reached a mean of 87% of the total population per arm.

**TABLE 1:** Population coverage and participation in the UPAVAN interventions^1^

Population	AGRI	AGRI-NUT	AGRI-NUT + PLA	Mean
Clusters	37	37	37	
Pregnant women and mothers of children age < 2 y in intervention clusters				
Total population (all ages) in intervention clusters	51,220	50,094	60,681	53,998
Coverage				
Primary intervention participants^2^	4389	4347	4965	4567
Number of women (any age) who participated	9202	9272	9626	9367
Participation				
Average number of participants per video dissemination group	20 (14–25)	19 (13–25)	18 (13–25)	19 (13–25)
Total group meetings attended by primary participants	61,446	60,858	44,685	55,663
Total home visits to primary intervention participants	59,482	57,051	33,052^3^	49,862
Total group meetings attended by all people	368,080	343,064	298,406	336,517
Total points of contact with all people^4^	427,562	400,115	331,458	386,378

1 Values are *n* or *n* (range). AGRI, nutrition-sensitive agriculture intervention; AGRI-NUT, nutrition-sensitive and nutrition-specific agriculture intervention; AGRI-NUT + PLA, nutrition-sensitive and nutrition-specific agriculture intervention using the participatory learning and action approach; PLA, participatory learning and action; UPAVAN: Upscaling Participatory Action and Videos for Agriculture and Nutrition. ^2^Primary intervention participants were pregnant women and mothers of children 0–23 mo of age who attended ≥1 dissemination group meeting and received a follow-up home visit.

3 Facilitators aimed to conduct home visits after every video dissemination group meeting but only after some PLA meetings, when appropriate, so fewer home visits were planned in the AGRI-NUT + PLA arm.

4 Total points of contact is sum of total dissemination meeting attendance by all people and total home visit.

### Program costs and program cost efficiency

Program costs are described in **Table 2** and presented by year in **Supplemental Material Table S4**. Total program costs of the AGRI, AGRI-NUT, and AGRI-NUT + PLA intervention arms were estimated as US$272,121, US$366,686, and US$386,907, respectively, and the mean annual costs of the intervention arms were estimated as US$79,645, US$107,323, and US$113,241, respectively. Start-up costs accounted for ∼26% of total costs, with the AGRI-NUT arm having the highest proportion of startup costs (30%) and the AGRI-NUT + PLA arm having the lowest (23%). The main reason for higher startup costs for the AGRI-NUT arm is that more staff time was spent preparing nutrition-specific videos and training, mainly driven by inputs from international staff.

**TABLE 2:** Cost description of the UPAVAN interventions from program perspective^1^

	AGRI		AGRI–NUT		AGRI–NUT + PLA	
Description	US$^2^	%	US$^2^	%	US$^2^	%
Total economic cost of program	272,121		366,686		386,907	
Startup costs	71,176	26%	108,978	30%	89,905	23%
Implementation costs	200,945	74%	257,708	70%	297,002	77%
Annual total economic costs^3^	79,645		107,323		113,241	
Annual implementation costs^4^	75,354	95%	96,641	90%	111,376	98%
Economic costs of main intervention components						
Developing and delivering NSA videos	186,912	69%	93,456	25%	93,456	24%
Developing and delivering nutrition-specific videos	0	0%	142,007	39%	47,336	12%
Running PLA sessions and developing nutrition-specific videos through PLA	0	0%	0	0%	61,557	16%
Quality assurance and monitoring activities	69,041	25%	101,976	28%	160,139	41%
Partner coordination	16,167	6%	29,247	8%	24,419	6%
Training government frontline workers^5^	4750	NA	4750	NA	4750	NA

1 AGRI, nutrition-sensitive agriculture intervention; AGRI-NUT, nutrition-sensitive and nutrition-specific agriculture intervention; AGRI-NUT + PLA, nutrition-sensitive and nutrition-specific agriculture intervention using the participatory learning and action approach; NA, not applicable; NSA, nutrition-sensitive agriculture; PLA, participatory learning and action; UPAVAN, Upscaling Participatory Action and Videos for Agriculture and Nutrition.

2 2019 US$.

3 Mean annual total intervention costs over a time horizon of 41 mo.

4 Mean annual total costs during implementation period over implementation period of 32 mo

5 These costs are not included in total program costs as they were implemented in all arms including the control arm.

**FIGURE 4:**
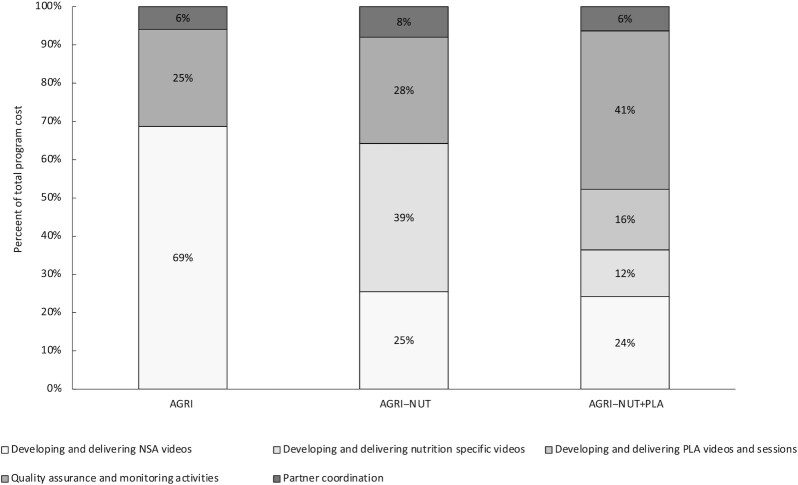
Composition of total program costs by UPAVAN intervention arm. AGRI, nutrition-sensitive agriculture intervention; AGRI-NUT, nutrition-sensitive and nutrition-specific agriculture intervention; AGRI-NUT + PLA, nutrition-sensitive and nutrition-specific agriculture intervention using the participatory learning and action approach; NSA, nutrition-sensitive agriculture; PLA, participatory learning and action; UPAVAN, Upscaling Participatory Action and Videos for Agriculture and Nutrition.

The main intervention activities of video production, group meetings, and follow-up home visits constituted the largest share of total costs (ranging from 52% in AGRI-NUT + PLA to 69% in AGRI). These activities were followed by the quality assurance and monitoring activities (ranging from 25% in the AGRI arm to 41% in the AGRI-NUT + PLA arm) (**Figure 4**). In the AGRI-NUT + PLA arm, more staff time was spent on the quality assurance of the PLA component from 1 partner (Ekjut).

Decomposing the total program costs to line items or inputs (as given in **Table 3**) shows that staff costs constituted the most costs, at ∼60% in each intervention arm, ranging from 56% in the AGRI-NUT arm to 65% in the AGRI-NUT + PLA arm. The staff costs were followed by other recurrent costs (travel costs and office overheads), varying from 29% in the AGRI-NUT + PLA arm to 39% in the AGRI-NUT arm, mainly due to the large portion of international travel by international staff (JSI RTI) in the AGRI-NUT arm. Most staff costs related to the delivery of interventions, i.e., salary for 24–26 facilitators per arm and 2–3 supervisors per arm, followed by support provided by technical assistance and research partners (**Supplemental Figure 1**). International staff costs were ∼33% of total staff costs, and mostly contributed to intervention development and technical support during implementation, particularly in the AGRI-NUT arm (Supplemental Figure 1).

**TABLE 3:** Total economic program costs from program perspective, by line item and UPAVAN intervention arm^1^

	AGRI		AGRI-NUT		AGRI-NUT + PLA	
Expenses	US$^2^	%	US$^2^	%	2019 US$^2^	%
Staff	162,153	60%	203,805	56%	249,794	65%
Materials	2848	1%	3445	1%	3159	1%
Capital	10,836	4%	16,539	5%	23,438	6%
Other recurrent	96,284	35%	142,897	39%	110,516	29%
Total	272,121		366,686		386,907	

1 AGRI, nutrition-sensitive agriculture intervention; AGRI-NUT, nutrition-sensitive and nutrition-specific agriculture intervention; AGRI-NUT + PLA, nutrition sensitive and nutrition-specific agriculture intervention using participatory learning and action approach; PLA, participatory learning and action; UPAVAN, Upscaling Participatory Action and Videos for Agriculture and Nutrition

2 2019 US$. An annual discount rate of 3% has been applied.

**Table 4** gives the cost per intervention from both program and societal perspectives, followed by the cost-efficiency estimates per primary participant and per point of contact. Program costs per primary intervention participant were US$62 in the AGRI arm, US$84 in the AGRI-NUT arm, and US$78 in the AGRI-NUT + PLA arm. Program costs per point of contact were US$0.64 in AGRI, US$0.92 in AGRI-NUT, and US$1.17 in AGRI-NUT + PLA.

**TABLE 4:** UPAVAN intervention costs and cost-efficiency by intervention arm^1^

Description	AGRI	AGRI-NUT	AGRI-NUT + PLA
Program costs and program cost-efficiency (US$)^2^			
Total cost	272,121	366,686	386,907
Total cost per primary intervention participant^3^	62	84	78
Annual cost per primary intervention participant^4^	18	25	23
Total cost per point of contact^5^	0.64	0.92	1.17
Societal costs, US$			
Total costs to the public healthcare providers	6125	4735	5025
Costs of increase in use of services	7793	1680	5096
Opportunity costs of involvement of frontline workers in interventions	3527	4174	3326
Total opportunity costs to the participants of attending the dissemination group meetings and home visits^6^	266,228	248,814	209,433
Total societal costs and cost efficiency, US$			
Program + provider costs	283,441	372,541	395,329
Societal costs (program + provider + participant)	549,668	621,355	604,762
Total societal cost per primary intervention participant	125	143	122
Total societal cost per points of contact	1.29	1.55	1.82

1 AGRI, nutrition-sensitive agriculture intervention; AGRI-NUT, nutrition-sensitive and nutrition-specific agriculture intervention; AGRI-NUT + PLA, nutrition sensitive and nutrition-specific agriculture intervention using participatory learning and action approach; PLA, participatory learning and action; UPAVAN, Upscaling Participatory Action and Videos for Agriculture and Nutrition

2 2019 US$.

3 Primary intervention participants were pregnant women and mothers of children 0–23 mo of age who attended ≥1 dissemination group meeting and received a follow-up home visit.

4 Mean annual total costs over a time horizon of 41 mo.

5 Total points of contact is sum of total dissemination meeting attendance and total home visits.

6 Included costs incurred by primary intervention participants and all other women who attended ≥1 dissemination group meeting.

### Societal costs of the interventions

Interventions slightly increased the use of maternal care services (such as delivery care, on-site feeding, and malaria testing) delivered by government frontline nutrition and health care providers, but participants did not incur significantly higher out-of-pocket expenditures. Based on the unit cost of these services, the total cost to the health system of this increased demand for health services ranged from US$1680 in the AGRI-NUT arm to US$7793 in the AGRI arm (Table 4).

Overall, government frontline nutrition and healthcare workers attended 9388 (in AGRI), 9509 (in AGRI-NUT), and 8012 (in AGRI-NUT + PLA) group meetings. Estimated total opportunity costs of their involvement ranged from US$3326 in the AGRI-NUT + PLA arm to US$4174 in the AGRI-NUT arm (Table 4).

Analyses of the endline survey data showed no evidence that the interventions increased participant household expenditures in total or on healthcare, agricultural inputs, food, or nonfood items (8). The estimated opportunity costs to the intervention participants from time spent participating ranged from US$209,433 in the AGRI-NUT + PLA arm to US$266,228 in the AGRI arm.

Taken together, total societal costs of the AGRI, AGRI-NUT, and AGRI-NUT + PLA interventions were US$549,668; US$621,355; and US$604,762; respectively. Total cost efficiency (program and societal costs per pregnant woman or mother of children aged 0–23 mo) was US$125 in AGRI, US$143 in AGR-NUT, and US$122 in AGRI-NUT + PLA. Total societal costs per point of contact were US$1.29, US$1.55, and US$1.82, respectively, (Table 4).

### Intervention consequences and cost–outcome results

Results in **Table 5** show that the AGRI-NUT and AGRI-NUT + PLA interventions increased the minimum dietary diversity of children, each compared with the control (adjusted RRs: 1.19 and 1.27; 95% CIs: 1.03, 1.37 and 1.11, 1.46, respectively. Both AGRI and AGRI-NUT + PLA increased minimum dietary diversity in mothers, each compared with the control [adjusted RR (95% CI): AGRI, 1.21 (1.01, 1.45); AGRI-NUT + PLA, 1.30 (1.10, 1.53)]. Furthermore, the AGRI intervention increased decision-making by women and the total and net annual value of agricultural production compared with the control arm. There was no statistically significant effect of the interventions on the other outcomes (8).

**TABLE 5:** UPAVAN intervention consequences, measured as effects on trial outcomes among trial participants included in the endline survey^1^

	Control	AGRI vs. control		AGRI-NUT vs. control		AGRI-NUT + PLA vs. control	
	*n*	*n*	aRR (95% CI)	*n*	aRR (95% CI)	*n*	aRR (95% CI)
Child minimum diet diversity (ate ≥4 food groups)	757	822	1.06 (0.91, 1.23),	812	1.19 (1.03, 1.37)^2^	863	1.27 (1.11, 1.46)^2^
Maternal minimum diet diversity (ate ≥5 food groups)	997	1100	1.21 (1.01, 1.45)^2^	1055	1.16 (0.98, 1.38)	1139	1.30 (1.10, 1.53)^2^
Child minimum acceptable diet	790	862	1.01 (0.86, 1.19)	829	1.19 (1.02, 1.41)^2^	895	1.30 (1.12, 1.52)
Women made ≥2 decisions in agriculture or health	997	1100	1.05 (1.00, 1.11)^2^	1055	1.05 (0.99, 1.1)	1139	1.02 (0.96, 1.07)
Total value of agricultural production over 1 y, US$	996	1100	108 (31, 231)^1^	1053	−5 (−134, 118)	1138	70 (−2, 190)
Net value (total value minus input costs) of agriculture production over 1 y, US$	996	1100	97 (25, 219)^2^	1053	−3 (−131, 112)	1138	76 (12, 196)

1 AGRI, nutrition-sensitive agriculture intervention; AGRI-NUT, nutrition-sensitive and nutrition-specific agriculture intervention; AGRI-NUT + PLA, nutrition-sensitive and nutrition-specific agriculture intervention using participatory learning and action approach; aRR, adjusted RR, only reported for the statistically significant outcomes of the trial; PLA, participatory learning and action; UPAVAN, Upscaling Participatory Action and Videos for Agriculture and Nutrition; vs., versus.

2 Significantly different from the control arm, *P* < 0.05.

**Table 6** presents results from our estimates for cost per mean change in maternal and child dietary diversity scores. The results show that, with US$287, the AGRI-NUT + PLA arm has the lowest cost per unit of improvement, reflecting the larger improvements in both maternal and child dietary diversity in the AGRI-NUT + PLA arm than in the AGRI and AGRI-NUT arms, both compared with the control arm.

**TABLE 6:** Cost outcome results from UPAVAN interventions, by intervention arm^1^

	AGRI	AGRI–NUT	AGRI–NUT + PLA
Total program cost, US$	272,121	366,686	386,907
Primary intervention participants covered,^2^*n*	4389	4347	4965
Mean child DDS^3^	0.0 (−0.15, 0.16)	0.13 (−0.04, 0.30)	0.28 (0.13, 0.44)^5^
Mean maternal DDS^4^	0.12 (−0.06, 0.30)	0.14 (−0.03, 0.31)	0.24 (0.08, 0.41)^5^
Cost per primary intervention participants, US$	62	84	78
Cost outcome, child DDS	—	603	278
Cost outcome, maternal DDS	517	603	325

1 AGRI, nutrition-sensitive agriculture intervention; AGRI-NUT, nutrition-sensitive and nutrition-specific agriculture intervention; AGRI-NUT + PLA, nutrition-sensitive and nutrition-specific agriculture intervention using participatory learning and action approach; DDS, dietary diversity score; PLA, participatory learning and action; UPAVAN, Upscaling Participatory Action and Videos for Agriculture and Nutrition.

2 Primary intervention participants (pregnant women and mothers of children <2 y of age who attended ≥1 women's group meeting). ^3^DDS: Dietary diversity score

4 One result from baseline missing due to implausible value.

5 Significantly different from the control arm, *P* < 0.05.

### Sensitivity and scenario analysis

**Table 7** shows that replacing international staff costs with local staff costs and reducing monitoring and startup costs had a large impact on the results. Replacing international staff costs with local staff costs reduced total costs by between 24% in the AGRI-NUT + PLA arm to 40% in the AGRI-NUT arm. Reducing costs of the monitoring information system by 50% (25%) reduced the total costs by between 13% (6%) in the AGRI arm to 21% (10%) in the AGRI-NUT + PLA arm. Reducing startup costs by 50% reduced the total costs and cost per primary intervention participant (pregnant women and mothers of children 0–23 mo of age) between 12% in the AGRI-NUT + PLA arm to 15% in the AGRI-NUT arm. Varying the discount rate or changing the allocation rule for the nutrition-specific component had a modest effect on the results, ranging from −10 to +10%.

**TABLE 7:** Results from sensitivity and scenario analyses by UPAVAN intervention arm^1^.

	Intervention arms					
	AGRI		AGRI–NUT		AGRI–NUT + PLA	
Scenarios/parameters	Total costs	Cost per primary intervention participant^3^	Total costs	Cost per primary intervention participant	Total costs	Cost per primary intervention participant
Base-case scenario	272,121	62	366,686	84	386,907	78
Allocation rule for nutrition-specific component between arms 2 and 3 (base-case 75% vs. 25%)						
Alternative allocation rule: 90% vs. 10%	272,121	62	403,726	93	349,867	70
Discount rate (base-case 3%)						0
Discount rate 0%	284,880	65	382,748	88	405,884	82
Discount rate 6%	260,470	59	352,000	81	369,612	74
Startup costs (base-case 100%)						
Reduce startup costs by 50%	236,533	54	312,197	72	341,955	69
MIS costs (base-case 100%)						
Reducing MIS costs by 25%	254,860	58	341,192	78	346,872	70
Reducing MIS costs by 50%	237,600	54	315,698	73	306,837	62
Replacing international costs with local staff	182,849	42	219,025	50	294,963	59

1 All values in 2019 US$. AGRI, nutrition-sensitive agriculture intervention; AGRI-NUT, nutrition-sensitive and nutrition-specific agriculture intervention; AGRI-NUT + PLA, nutrition-sensitive and nutrition-specific agriculture intervention using participatory learning and action approach; MIS, monitoring information system; PLA, participatory learning and action; UPAVAN, Upscaling Participatory Action and Videos for Agriculture and Nutrition; vs., versus.

2 Program costs

3 Primary intervention participants were pregnant women and mothers of children aged 0–23 mo who attended ≥1 dissemination group meeting.

### Cost and affordability of scaleup

Given that AGRI-NUT + PLA was the only intervention to increase both maternal and child dietary diversity, we modeled the potential cost of scaling up the AGRI-NUT + PLA intervention to all rural districts in Odisha. The cost would be ∼US$65 million per y, based on a cost of US$1.9 per person (total population in the AGRI-NUT + PLA intervention clusters). This is ∼5% of the proposed state health budget for 2021–2022 (∼US$1.3 billion per y) or 1.7% of the combined health and agriculture budget (43). However, unit costs might be lower due to potential economies of scale. In addition, given the investments already made in developing the UPAVAN interventions, it is expected that monitoring and evaluation, startup, and coordination activities will be less intensive at scale, reducing implementation costs significantly, as shown in the sensitivity and scenario analyses.

## Discussion

This study contributes to the limited economic evaluation evidence on multisectoral nutrition interventions and is to our knowledge the first economic evaluation of a nutrition-sensitive agriculture intervention in South Asia. We found that participatory NSA interventions, with different combinations of nutrition-specific behavior change or PLA components, can increase child and maternal minimum dietary diversity. The total costs of designing and implementing the UPAVAN interventions ranged from US$271,121 to US$386,907, and annual costs ranged from US$79,645 to US$113,241. Throughout the 32 mo of implementation, the interventions covered a mean of 4567 pregnant women and mothers of children 0–23 mo of age per intervention, and the cost per pregnant woman or mother (the primary intervention participant) ranged from US$62 in the AGRI arm to US$84 in the AGRI-NUT arm.

Making comparison between UPAVAN and other NSA interventions is challenging due to differences in intervention components, delivery platforms, scales, outcomes assessed, and costing approaches. However, 2 interventions share some similarities with the UPAVAN interventions: NEEP-IE (Nutrition Embedded Evaluation Programme Impact Evaluation) in Malawi (11, 12, 44), and Mama SASHA (Sweetpotato Action for Security and Health in Africa) in Kenya (10). NEEP-IE integrated NSA with nutrition-specific training and used community-based early childhood development centers and parenting group platforms (44, 45). Mama SASHA promoted production and consumption of orange-fleshed sweet potato and integrated nutrition-specific and health components, delivered through health facilities, community health workers, and extension officers (46). UPAVAN, NEEP-IE, and Mama Sasha had similar cost efficiency, at US$62 to US$84 per participant in UPAVAN, US$160 per preschool child covered in NEEP-IE (11), and US$110 per woman and child covered in Mama SASHA (10). In all 3 programs, staff and travel costs constituted major shares (UPAVAN: 56–60%; NEEP-IE: 40%; Mama SASHA: 25%). Comparing point estimates, we found that, for child dietary diversity, AGRI and AGRI-NUT were less cost-effective than NEEP-IE and Mama SASHA, but AGRI-NUT + PLA was more cost-effective (AGRI had no effect; cost per food group increase for AGRI-NUT: US$603; NEEP-IE: US$444; Mama SASHA: US$305; and AGRI-NUT + PLA US$278). Similarly, AGRI and AGRI-NUT were less cost-effective than Mama SASHA at improving maternal diet diversity, but AGRI-NUT + PLA was more cost-effective (cost per food group increase for: AGRI US$517; AGRI-NUT US$603; Mama SASHA US$324; AGRI-NUT + PLA US$325) (47). For NEEP-IE, effects on maternal diet diversity were not reported.

Taken together, our findings show broadly similar cost profiles, cost-efficiency, and cost-effectiveness for UPAVAN, NEEP-IE, and Mama SASHA. Although the UPAVAN trial was not designed to detect differences between intervention arms, we note that the higher cost-effectiveness in the AGRI-NUT + PLA intervention compared with that in the control arm suggests that the AGRI-NUT + PLA intervention is a better value for the money than the AGRI or AGRI-NUT interventions. Therefore, the Participatory Learning and Action component may help to increase cost-effectiveness of NSA interventions. The choice of which intervention to scale up will depend on transferability of intervention models across contexts, and policymaker priorities for other outcomes. For example, NEEP-IE had additional effects on stunting [with cost-effectiveness ratios of US$595 per case of stunting averted, and US$516 per disability-adjusted life years (DALYs)averted (12)], and both NEEP-IE and AGRI interventions increased agricultural production, whereas Mama SASHA specifically increased production of orange-flesh sweet potatoes and nutrition knowledge.

We consider how NSA interventions compare to other nutrition-specific interventions, beyond the 10 nutrition-specific interventions already recommended at scale (at a cost of US$125 to US$571 per life-year saved). Nutrition-specific women's groups using a PLA approach (48) and SHG (49) have shown similar cost efficiency (US$62 to US$140 per participant), and similar magnitude of effects on dietary diversity of mothers [adjusted OR >5/10 food groups: 1.4 (48)], and children [mean difference: 0.17 food groups (49)]. Biofortification with nutrition education is less expensive, at US$65 and US$49 per beneficiary household in Mozambique and Uganda, respectively, and can double vitamin A intakes in children (50). A modeling study of food-based interventions showed that, in India, mass media campaigns and complementary food processing interventions would be more cost effective than a household horticulture intervention (US$90 and US$41, compared with US$644 per life-year saved) (51).

We conclude that NSA interventions have cost efficiency and effectiveness similar to those for nutrition-specific women's group interventions but may be less cost effective at improving nutrition than other approaches, such as mass media campaigns, complementary food processing, and food fortification. However, NSA can have added benefits of improved agricultural productivity, which nutrition-specific interventions would be less likely to achieve. In contrast, addition of agricultural components to nutrition and health education interventions, such as home-gardens (52) and community vegetable gardens (53), is prohibitively expensive, at ∼2014 US$918 per beneficiary in Bangladesh (52) and EUR 1525 per beneficiary household in Zimbabwe (53).

### Policy implications: scalability and affordability

Before we conducted the trial, some components of the UPAVAN interventions were already being implemented at scale in several states in India. Participatory agriculture extension videos have been scaled up across several states in India, in collaboration with National and State Livelihood Missions and with the use of a women's self-help group platform reaching ∼2 million smallholder farmers (54). Participatory nutrition-specific videos have been scaled up across 5 states in India, in collaboration with State Livelihood Missions, the National Health Mission, and other state-level institutions, reaching around half a million pregnant women and mothers of children aged 0–23 mo (55). In addition, supported by the National Health Mission, PLA groups have been scaled up across all districts in Jharkhand and one-third of districts in the Madhya Pradesh states, through incentivized ASHAs and ASHA supervisors (56, 57). Evidence on statewide scaleups of PLA groups in Jharkhand showed that employing incentivized ASHAs and ASHA supervisors and using an innovative approach for training at scale resulted in a substantial reduction in implementation costs, without compromising the impact of the intervention (58, 59). Similar models could be tested for participatory video–based NSA interventions.

Although there is clearly policy interest in the UPAVAN components, their implementation at scale is currently only being conducted by government frontline workers from single sectors. Scaleup of the UPAVAN interventions and other multisectoral approaches will require coordination of multiple stakeholders across sectors, for which the challenges are well recognized (12). It is hoped that our economic evaluation will provide further impetus to increase collaboration across sectors, but stakeholder analyses may be needed to further support this goal.

### Limitations

Some limitations of this study are notable. First, adoption of practices promoted by the UPAVAN interventions, or collective actions taken by participating communities, might have caused direct and indirect costs and benefits to participants and the communities that were not accounted for. As with any complex economic evaluation, it is difficult to determine the direction of bias in our overall conclusions because it was also not possible to fully identify and measure all of the benefits associated with the interventions. The trial measured only effects on children 0–23 mo of age and their mothers and families and did not measure effects on the communities as a whole. The trial also did not include other possible short- and long-term consequences for participants, such as improvements in soil quality, micronutrient adequacy, intrahousehold relationships, social support, mental health, or overall wellbeing. In addition, some community members and government frontline health and nutrition care workers were involved in the production of NSA and nutrition-specific videos. The costs of their involvement have not been included here but are likely to have been small and would not have seriously affected the study findings. Third, as is standard practice, the trial was powered to detect differences only in the primary outcomes. Statistically insignificant effects in other outcomes, such as household food expenditures and healthcare seeking or utilization may have been due to lack of statistical power rather than lack of effect. Finally, we do not report cost-effectiveness in terms of outcomes commonly used in cost-effectiveness analyses of nutrition interventions (such as DALYs or life-years saved), which limits comparisons with other nutrition interventions. The outcomes that the interventions aimed to change cannot be easily translated to DALYs or other common outcomes for.

## Conclusions

In this study we estimated the cost of designing and implementing 3 participatory NSA interventions in rural India, responding to the gap in evidence on the costs of multisectoral NSA interventions and providing useful data to inform their potential scaleup. Our findings show that the costs per primary intervention participant of implementing the interventions are comparable with the results from a limited set of evaluated multisectoral NSA interventions. Considering that a substantial investment has already been made to develop the UPAVAN interventions, costs at scale could be decreased with a less intensive information monitoring system, reduced startup costs, integration within existing programs, and possible economies of scale. We recommend scaleup of AGRI-NUT + PLA, which had the lowest cost per unit increase in dietary diversity. This scaleup should be feasible, given that participatory videos on agriculture, nutrition, and PLA groups are already being implemented at scale in several settings, and because the intervention approach is designed to be responsive to local contexts.

## Supplementary Material

nxac132_Supplemental_Files

## Data Availability

Aggregated cost data are provided in the tables within paper and in supplemental tables. Trial outcome data will be available from the LSHTM Data Compass, an open-assess institutional research data repository, at https://datacompass.lshtm.ac.uk/.

## Notes

The UPAVAN trial was funded by the Bill & Melinda Gates Foundation and UK Aid (award number OPP1136656). Substantial cofunding was also provided by the USAID-funded project Digital Integration to Scale Gender-Sensitive Nutrition Social and Behavior Change Communication, implemented by Digital Green (Cooperative Agreement No. AID-386-A-15–00008). Additional technical inputs were provided by the SPRING project contributing funds from USAID's Bureau for Food Security (BFS) and Bureau for Global Health (GH) (under Cooperative Agreement No. AID-OAA-A-11-00031). The trial funders had no role in study design, data collection, analysis, interpretation or writing of the paper.

Author disclosures: The authors report no conflicts of interest.

Supplemental Material Tables S1–S4, Supplemental Material–data collection tools, Supplemental Material - Sensitivity and scenario analyses, and Supplemental Figure 1 are available from the “Supplementary data” link in the online posting of the article and from the same link in the online table of contents at https://academic.oup.com/jn/.

HH-B and HH-F are joint first authors.

Abbreviations used: ASHA, accredited social health activist; JSI RTI, John Snow Inc. Research and Training Institute; LSHTM, London School of Hygiene & Tropical Medicine; Mama SASHA, Sweetpotato Action for Security and Health in Africa; NEEP-IE, Nutrition Embedded Evaluation Programme Impact Evaluation; NSA, nutrition-sensitive agriculture; PLA, participatory learning and action; SHG, self-help group; UCL, University College London; UPAVAN, Upscaling Participatory Action and Videos for Agriculture and Nutrition; VARRAT, Voluntary Association for Rural Reconstruction and Appropriate Technology; DALYs, Disability-Adjusted Life Years
