# Supplementary material for: Economic Evaluation of Nutrition-Sensitive Agricultural Interventions to Increase Maternal and Child Dietary Diversity and Nutritional Status in Rural Odisha, India
Source: J Nutr. 2022 Jun 10;152(10):2255–68. doi: 10.1093/jn/nxac132 (PMC9535442; doi:10.1093/jn/nxac132)
Supplement: nxac132_Supplemental_Files [file nxac132_supplemental_files.zip › Supplemental Materials-merged.docx]

**Data collection tools**

Definitions

**
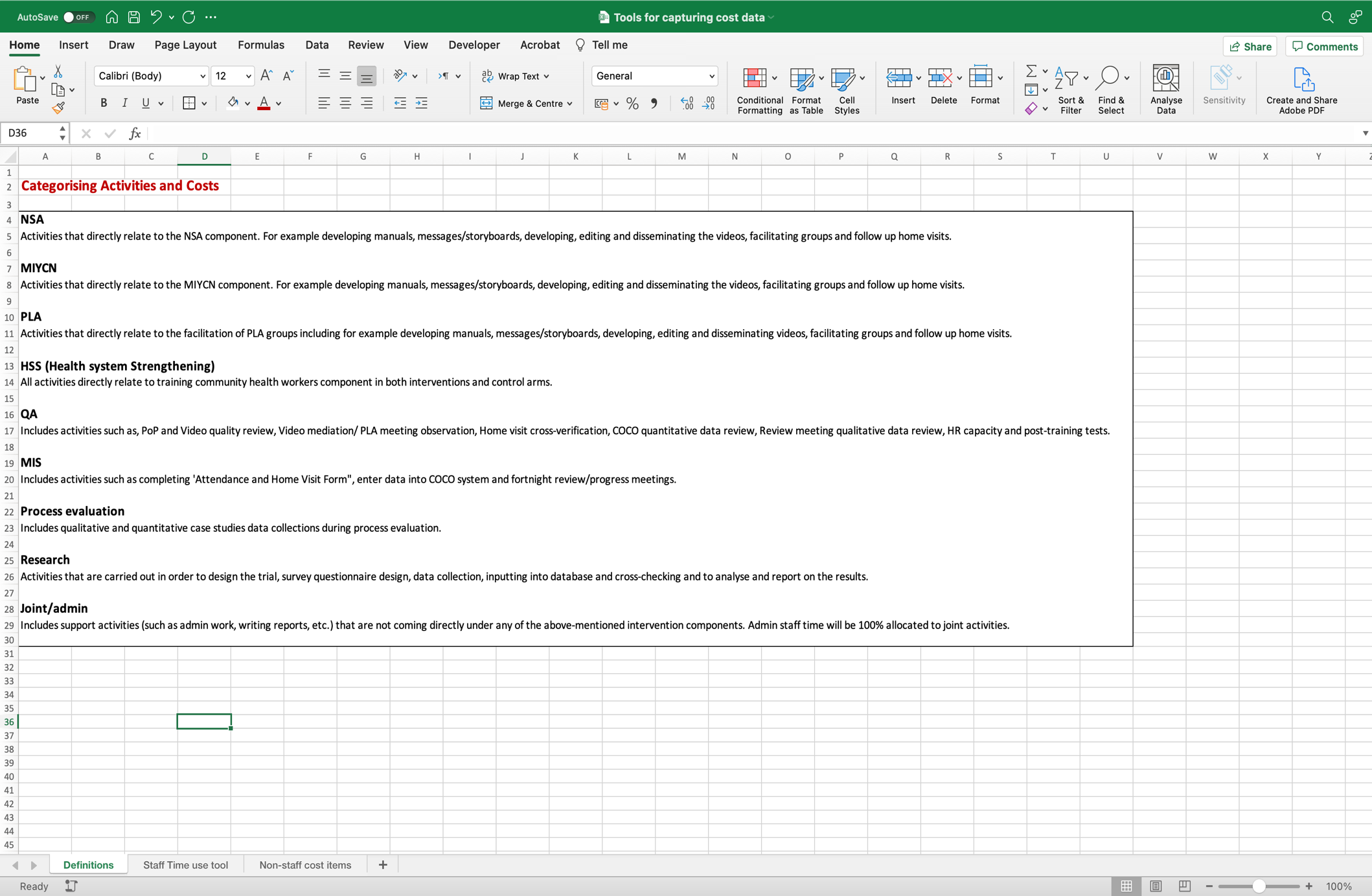
**

**Data collection tools-continue**

Staff Time use tool

**
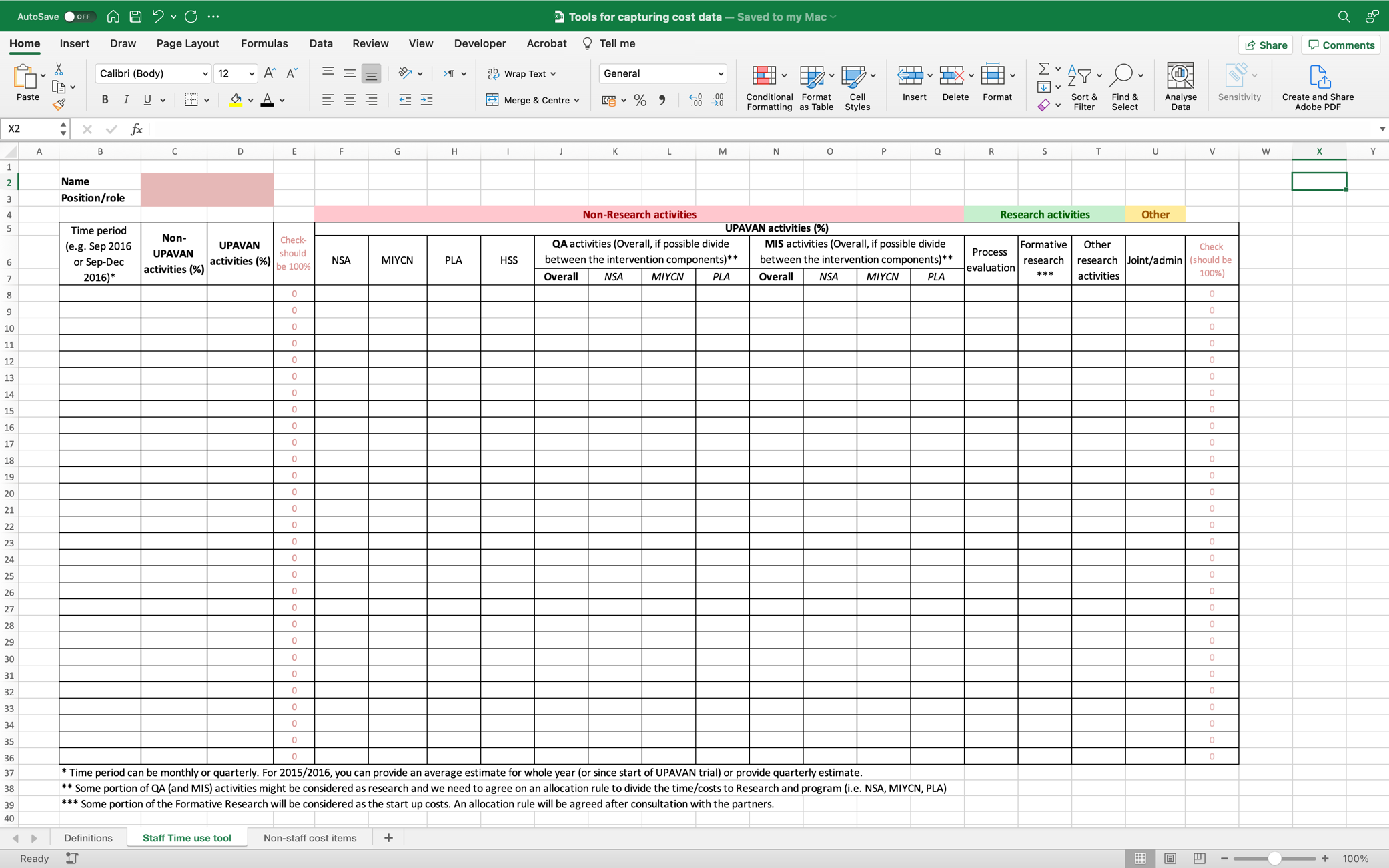
**

**Data collection tools-continue**

Non-staff cost items

**
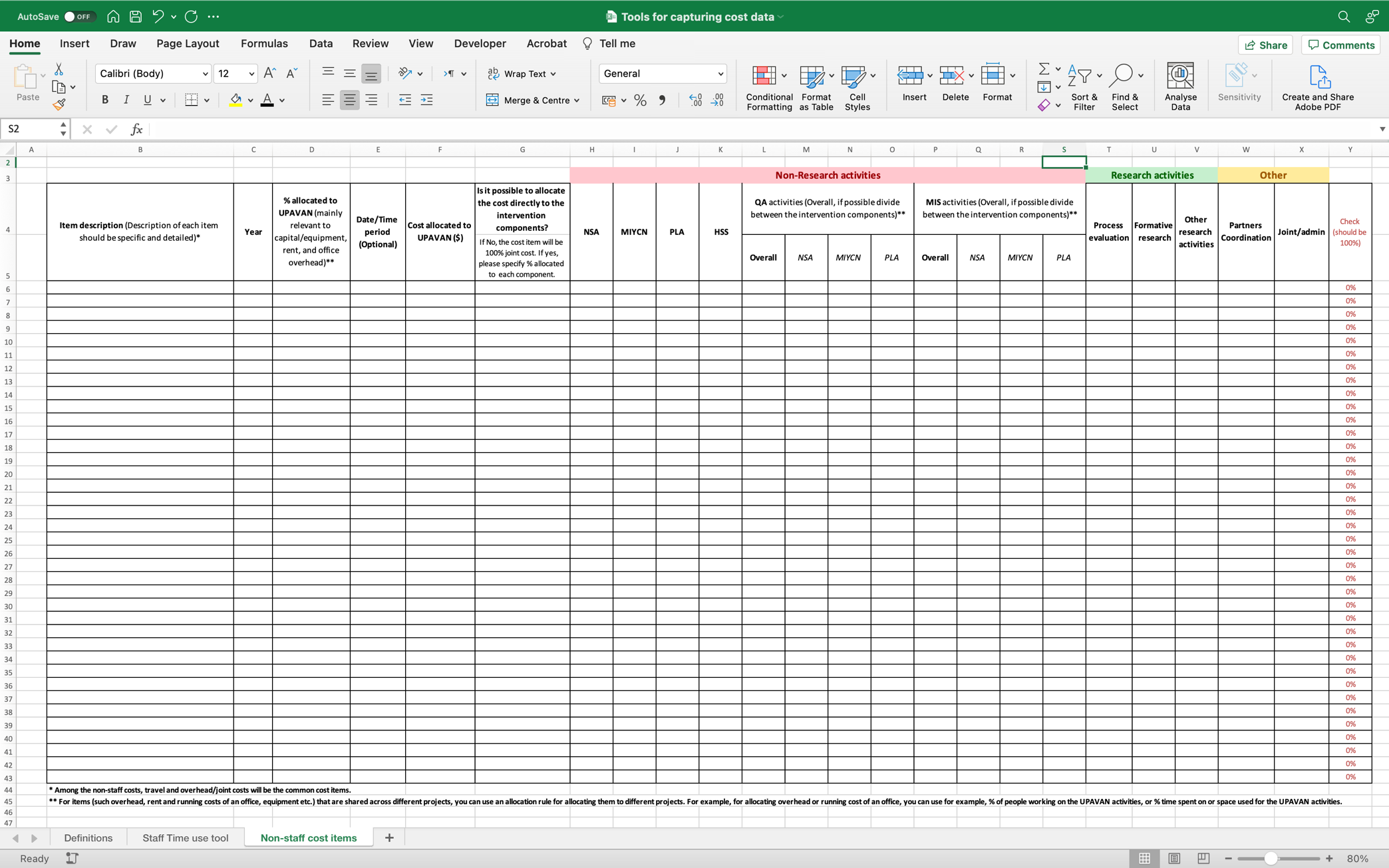
**

Table S1: Detailed description of outcome measures used in UPAVAN trial ^1^

| Outcome | | Indicator | | |
| --- | --- | --- | --- | --- |
| Primary outcomes | | | | |
| Child dietary diversity | | - Proportion of children 6-23 months of age consuming ≥4 food out of 7 groups in the previous 24 hours (assessed by 24-hour recall, using WHO-defined food groups) ^2^ | | |
| Maternal underweight | | - Mean body-mass index (BMI) (kg/m^2^) of non-pregnant, non-postpartum (gave birth >42 days ago) mothers or female primary caregivers of children aged 0 to 23 months | | |
| Secondary outcomes | | | | |
| Maternal dietary diversity | | - Proportion of mothers or female primary caregivers consuming ≥5 food out of 10 groups in the previous 24 hours (assessed by 24-hour dietary recall using FAO-defined food groups)^3^ | | |
| Child wasting | | - Proportion of children aged 0 to 23 months who are wasted (weight-for-height < -2 SD of WHO growth standards median)^4^ | | |
| Other outcomes | | | | |
| Maternal wasting | | - Proportion of pregnant and non-pregnant mothers or female primary caregivers with mid-upper arm circumference <230 mm^5^ | | |
| Child acute malnutrition | | - Proportion of children (aged 6-23 months) with acute malnutrition (mid-upper arm circumference <125mm)^4^ | | |
| Maternal and child haemoglobin (Hb) concentrations | | - Mean haemoglobin concentration (g/dl) of children (6-23 months) - Mean haemoglobin concentration (g/dl) of non-pregnant mothers or female primary caregivers | | |
| Infant and young child feeding (IYCF) practices | | - Proportion of children (aged 6-23 months) receiving the WHO-recommended ‘Minimum Acceptable Diet’^6^ | | |
| Women’s decision making | | - Proportion of women ‘empowered’ in decision-making in agriculture and health related domains (made >=2 decisions), measured using the Women’s Empowerment in Agriculture Index^7^ | | |
| Women’s time use | | - Proportion of women ‘empowered’ in the women’s time use domain (worked <10.5 hours in the previous 24 hours), measured using the Women’s Empowerment in Agriculture Index | | |
| Gender parity in agriculture | | - Proportion of women achieving gender parity between themselves and a male household member, measured using the Women’s Empowerment in Agriculture Index^7^. Gender parity achieved when women have equal or higher empowerment scores than men, and empowerment scores are calculated as weighted sums of five indicators: decision making, asset ownership, access to credit, group membership, and time use. Gender parity was measured in 50% of households and excludes female-only households. | | |
| Household economic status and food security | | - Mean per capita household share of food expenditures. Measured in 50% of households. - Mean per capita total daily household expenditure (INR). Measured in 50% of households. | | |
| Household agriculture production | | - Mean production diversity over the previous year (count out of 10 food groups produced, using FAO-defined food groups)^8^ - Total value of agricultural production over the previous year (INR) - Net value of agricultural production (total value of agricultural production minus input costs) over the previous year, (INR). | | |

**Table S2: Estimation of UPAVAN costs**

|  | **Cost type** | **Data source** | **Assumptions or details** |
| --- | --- | --- | --- |
| **Program costs** | | | |
| Direct | Financial cost data | Expenditure records and project accounts. | Capital costs annualized based on the estimated lifetime of each item, using a discount rate of 3%. |
|  |  | Staff time use data used to allocate staff costs and other joint costs to cost centers |  |
| Indirect | Value of donated items, mainly video-making and editing equipment | Items identified and market value assessed from staff interviews | Discounted value of donated items: US$ 1,455 |
|  | Volunteered time, mainly time on design of intervention materials by technical partners | Donated time assessed from staff time use surveys | Value of volunteer time: US$ 35,307 |
|  |  | Opportunity cost of time from staff monthly salaries from project accounts |  |
| **Costs to health system** | | | |
| Direct | Cost to health system of increase in demand for health and nutrition services | Difference in % households using any health or nutrition services in the past 18 months, in each intervention relative to control, assessed from endline survey | On average 4% increase in few selected maternal and child services. |
|  |  | Published data on unit cost of to health center of providing health and nutrition services (31-36). |  |
| Indirect | Opportunity cost of attending UPAVAN group meetings by government frontline health and nutrition workers | Attendance from facilitators’ registers | Meetings attended by ANMs   - AGRI: 286 - AGRI-NUT: 579 - AGRI-NUT+PLA: 110.   Meetings attended by ASHAs   - AGRI: 4802 - AGRI-NUT: 4264 - AGRI-NUT+PLA: 2874   Meetings attended by AWW   - AGRI: 4300 - AGRI-NUT: 4666 - AGRI-NUT+PLA: 5028 |
|  |  | Time spent attending meetings, assessed in endline survey | Mean meeting duration: 64 minutes |
|  |  | Time spent travelling to meetings, assessed in endline survey | Mean travel time: 9 minutes |
|  |  | Opportunity cost of time, as published salaries | Monthly salaries of:   - ANM: INR 25,000 (US$355) (1) - AWW: INR7,500 (US$106.5) (2); - ASHA: INR2000 (US$28.4) (3) |
| **Cost to participants** | | | |
| Indirect | Opportunity cost of participating | Participant attendance from facilitators’ registers | Total number of participants, summed over all meetings:   - AGRI: 368,080 - AGRI-NUT: 343,064 - AGRI-NUT+PLA: 298,406 |
|  |  | Time spent attending meetings, assessed in endline survey | Mean meeting duration: 64 minutes |
|  |  | Time spent travelling to meetings, assessed in endline survey | Mean travel time: 9 minutes |
|  |  | Number of home visits, from facilitators’ home visiting forms | - AGRI: 59482 - AGRI-NUT: 57,051 - AGRI-NUT+PLA: 33,052 |
|  |  | Time spent on home visits, assessed in endline survey | Mean home visit duration: 47 minutes |
|  |  | Opportunity cost of time, as published minimum daily wage of an agricultural worker in Odisha state | 303 Indian Rupees or US$4.31 ^9^ |
|  | Cost of adopting promoted practices | Difference between intervention and control in expenditures on healthcare (out-of-pocket fees and travel time), agricultural inputs, food and non-food costs, assessed using the expenditure survey in the endline survey. | No significant difference |

ANM: Auxiliary Nurse Midwife ; ASHA: Accredited Social Health Activist ;  AWW: Anganwadi worker

AGRI: Nutrition-sensitive agriculture intervention; AGRI-NUT: Nutrition-sensitive and nutrition-specific agriculture intervention; AGRI-NUT+PLA: Nutrition sensitive and nutrition-specific agriculture intervention using Participatory Learning and Action approach; UPAVAN: Upscaling Participatory Action and Videos for Agriculture and Nutrition

Table S3: Intervention costs by input/line items and intervention component: definitions

|  | Description |
| --- | --- |
| Costs by input/line item | |
| Staff | Value of staff time contributed to development and implementation of the interventions. Staff included group facilitators, facilitators’ supervisors, video producers, and technical staff (both within and outside India) |
| Materials | Costs of diaries for groups facilitators, manual and picture cards printing (mainly related to PLA meetings), and stationary and other printing costs |
| Capital | Costs of equipment for developing and disseminating videos, such as camera, video camera, projectors, laptop/PC, printer, as well as and field offices' equipment and furniture |
| Other recurrent | Mainly included travel costs, specifically, travel allowances for group facilitators and video developers, field travel costs for supervisors and other implementation and technical staff. It also includes overhead costs such as office rent and running costs. |
| Costs by intervention component | |
| AGRI- nutrition sensitive agriculture (NSA) videos | Included costs of technical support in developing content for NSA videos, shooting and editing the videos (includes salary for video developers), and running the dissemination meetings (mainly, salary for facilitators and supervisors) |
| NUT- nutrition specific videos | Included costs of technical support in developing content for nutrition specific videos, shooting and editing the videos (includes salary for video developers), and running the dissemination meetings (mainly, salary for facilitators and supervisors) |
| PLA | Included costs of technical support in developing and adapting PLA materials, shooting and editing the videos (includes salary for video developers), and running the PLA meetings (mainly, salary for facilitators and supervisors and other costs of running PLA groups including picture card and module printing) |
| Quality assurance and MIS | Includes costs of setting up monitoring information system, data entry, video quality and content checks, and monthly review meetings |
| Coordination | Coordination activities occurred mainly by one of technical partner (Digital Green) and included staff time contribution, travel costs and coordination meetings between implementing and technical partners. |
| Training government frontline workers | Included costs of two-day training in maternal, infant and young child nutrition to government frontline nutrition and health workers in all trial arms. Costs included travel allowance, siting fees, handouts, and value of staff time contribution. |
| Costs by implementation phase | |
| Start up | Costs of activities occurred during 9 months start up or preparation period.  Main activities during the start-up period included recruitment and training of group facilitators and their supervisors, community sensitisation activities, as well as development of video content. |
| Implementation | All costs occurred during 32 months of interventions implementation or running group meetings. |

**Sensitivity and scenario analyses**

We conducted following univariate sensitivity analyses that vary one parameter at a time, to examine the effects of uncertain assumptions on our results.

**First**, we used an alternative allocation rule for dividing the costs of the nutrition-specific component between the AGRI-NUT and AGRI-NUT+PLA arms. Except for the implementing partner, all other partners provided technical support to a specific intervention component. We therefore used an allocation rule, primarily based on the number of videos shown (and the content of the meetings, in the case of PLA), to allocate component costs between trial arms. The allocation rule used in base-case estimates was: in the AGRI arm, 50% of NSA component costs; in the AGRI-NUT arm, 25% of NSA component costs + 75% of nutrition-specific component costs; in the AGRI-NUT+PLA arm, 25% of NSA component costs + 25% of nutrition-specific component costs + 100% PLA component costs. In our sensitivity analysis, we used an alternative allocation rule of 90% and 10% to allocate costs of the nutrition-specific components to AGRI-NUT and AGRI-NUT+PLA, respectively. These allocation ratios were chosen based on consultation with VARRAT, Ekjut, Digital Green and JSI RTI.

**Second**, as recommended by economic evaluation guidelines ^10, 11^, alternative discount rates of 0% and 6% were used in the sensitivity analysis.

We also conducted the following three scenarios that are applicable to potential scale up of the interventions and guided by recent scale up of a similar intervention in India ^12^ and by experience of our implementing partners in India.

**Scenario 1**: start-up costs were around 26% of total intervention costs and included activities such as recruitment and training the facilitators (n=75 in total), community approvals and sensitizations, and development and adaptation of the intervention components (which heavily involved international staff). Outside of a trial setting, the start-up period will be slightly shorter and mainly focus on adaptation of the interventions for scale-up or to an alternative context. Taking this into consideration, start-up costs were reduced by 50% as an alternative scenario.

**Scenario 2:** a customized MIS was developed for UPAVAN interventions to capture data on group attendance, knowledge recall, and practice adoption, which was a resource intensive system. A scaled version of the interventions would not require this level of intensity and for this reason, 25% and 50% reductions in the cost of the monitoring and data collection system were considered as alternative scenarios, in consultation with the implementation partners.

**Scenario 3:** international staff (LSHTM, UCL and JSI RTI) time contribution to developing interventions, training and quality assurance, as well as other direct and indirect costs were included in the interventions’ costs. At scale, these interventions will be delivered by local organizations. Therefore, as an alternative scenario, local staff salaries were used instead of international staff salaries and equivalent local costs were used for international travel costs and indirect costs.

Table S4: Total program cost^1^ by UPAVAN arm and year (US$^2^)

| Arm | 2019 | 2018 | 2017 | 2016 | Total |
| --- | --- | --- | --- | --- | --- |
| AGRI | 53,867 | 72,888 | 110,847 | 34,519 | 272,121 |
| AGRI+NUT | 63,761 | 90,820 | 153,924 | 58,181 | 366,686 |
| AGRI+NUT+PLA | 85,088 | 112,932 | 140,297 | 48,590 | 386,907 |

^1^ An annual discount rate of 3% has been applied.

^2^ 2019 US$

AGRI: Nutrition-sensitive agriculture intervention; AGRI-NUT: Nutrition-sensitive and nutrition-specific agriculture intervention; AGRI-NUT+PLA: Nutrition sensitive and nutrition-specific agriculture intervention using Participatory Learning and Action approach; UPAVAN: Upscaling Participatory Action and Videos for Agriculture and Nutrition

**References**

1. Kadiyala S, Prost A, Harris-Fry H, et al. Upscaling Participatory Action and Videos for Agriculture and Nutrition (UPAVAN) trial comparing three variants of a nutrition-sensitive agricultural extension intervention to improve maternal and child nutritional outcomes in rural Odisha, India: study protocol for a cluster randomised controlled trial. *Trials* 2018; 19: 176. DOI: 10.1186/s13063-018-2521-y.

2. WHO. *Indicators for assessing infant and young child feeding practices part 2: measurement.* Geneva, Switzerland: WHO, 2010.

3. FAO and FHI 360. *Minimum Dietary Diversity for Women:A Guide for Measurement*. 2016. Rome: the Food and Agriculture Organization of the United Nations.

4. WHO and UNICEF. *WHO child growth standards and the identification of severe acute malnutrition in infants and children: A Joint Statement*. 2009.

5. Tang AM, Dong K, Deitchler M, et al. *Use of Cutoffs for Mid-Upper Arm Circumference (MUAC) as an Indicator or Predictor of Nutritional and HealthRelated Outcomes in Adolescents and Adults: A Systematic Review*. 2013. Washington, DC: FHI 360/FANTA.

6. WHO. *Indicators for assessing infant and young child feeding practices: definitions and measurement methods*. 2021. World Health Organization.

7. Malapit HJ, Kovarik C, Sproule K, et al. *Instructional guide on the abbreviated Women’s Empowerment in Agriculture Index (A-WEAI)*. 2015. Washington, D.C.: International Food Policy Research Institute (IFPRI).

8. Sibhatu KT, Krishna VV and Qaim M. Production diversity and dietary diversity in smallholder farm households. *Proc Natl Acad Sci U S A* 2015; 112: 10657-10662. 2015/08/12. DOI: 10.1073/pnas.1510982112.

9. Minimum Wage – Odisha, <https://paycheck.in/salary/minimumwages/19709-odisha/19710-agriculture> (2020).

10. Tan-Torres Edejer T, Baltussen R, Adam T, et al. *Making choices in health: WHO guide to cost-effectiveness analysis*. Geneva: World Health Organization, 2003.

11. Claxton K, Revill P, Sculpher M, et al. *The Gates Reference Case for Economic Evaluation*. 2014. The Bill and Melinda Gates Foundation.

12. Nair N, Tripathy PK, Gope R, et al. Effectiveness of participatory women's groups scaled up by the public health system to improve birth outcomes in Jharkhand, eastern India: a pragmatic cluster non-randomised controlled trial. *BMJ Glob Health* 2021; 6 2021/11/05. DOI: 10.1136/bmjgh-2021-005066.
