# Supplementary figures and images for: Economic Evaluation of Nutrition-Sensitive Agricultural Interventions to Increase Maternal and Child Dietary Diversity and Nutritional Status in Rural Odisha, India
Source: J Nutr. 2022 Jun 10;152(10):2255–68. doi: 10.1093/jn/nxac132 (PMC9535442; doi:10.1093/jn/nxac132)

**Supplementary Figure 1: Disaggregation of the staff costs across all UPAVAN interventions**

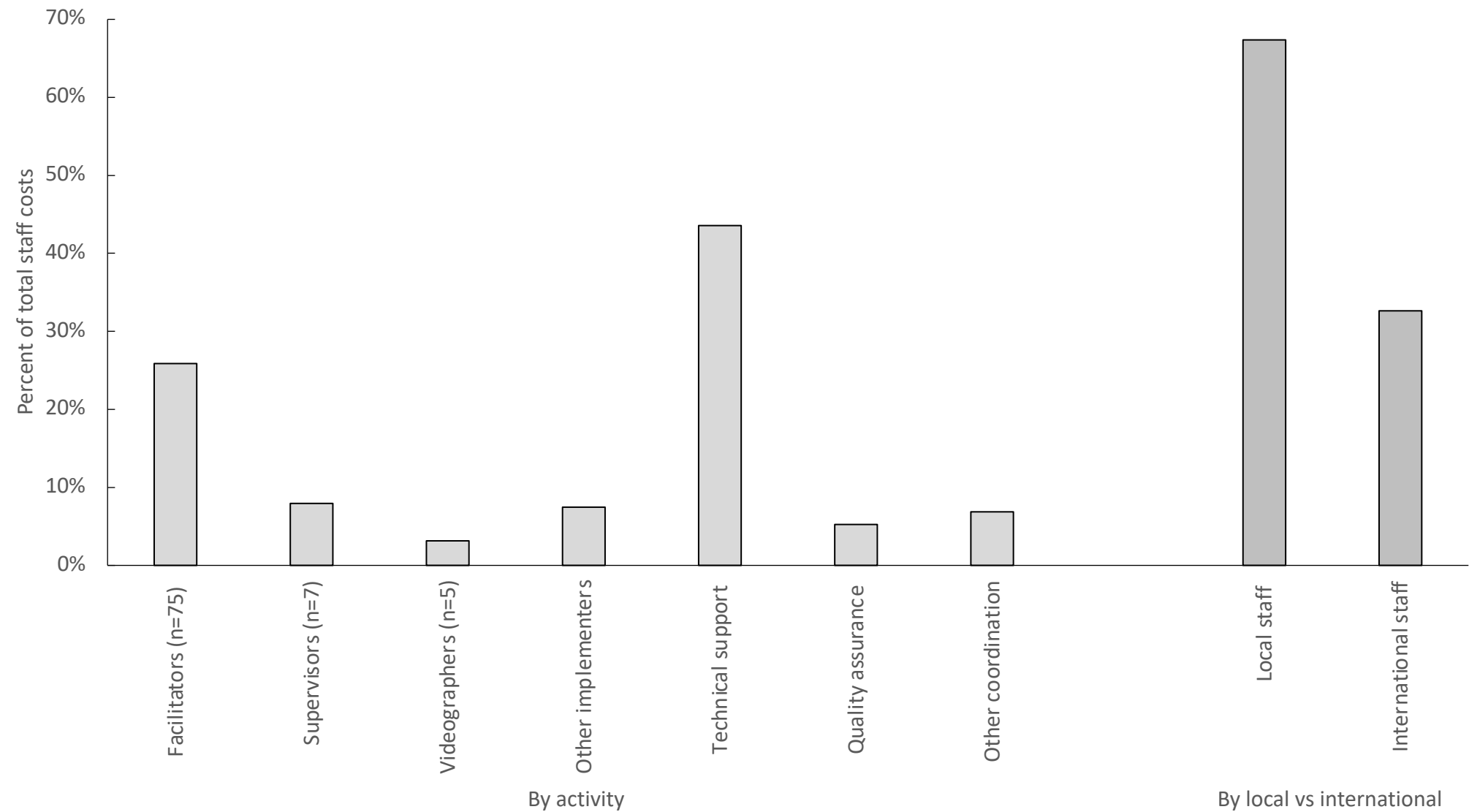

Supplement: nxac132_Supplemental_Files [file nxac132_supplemental_files.zip › Supplemental Figure 1.pdf]
